# Fermented Dessert with Whey, Ingredients from the Peel of Jabuticaba (*Myrciaria cauliflora*) and an Indigenous Culture of *Lactobacillus plantarum*: Composition, Microbial Viability, Antioxidant Capacity and Sensory Features

**DOI:** 10.3390/nu10091214

**Published:** 2018-09-02

**Authors:** Maria Carmélia Almeida Neta, Anna Paula Rocha de Queiroga, Raphael Lucas Jacinto Almeida, Anderson Caetano Soares, Jade Marinho Gonçalves, Suenia Soares Fernandes, Marina Cínthia de Sousa, Karina Maria Olbrich dos Santos, Flávia Carolina Alonso Buriti, Eliane Rolim Florentino

**Affiliations:** 1 Programa de Pós-Graduação em Ciências Farmacêuticas, Centro de Ciências Biológicas e da Saúde, Universidade Estadual da Paraíba, 58429-600 Campina Grande, PB, Brazil; carmelianeta20@hotmail.com (M.C.A.N.); marinacinthia@hotmail.com (M.C.d.S.); elianerf@yahoo.com.br (E.R.F.); 2 Núcleo de Pesquisa e Extensão em Alimentos, Centro de Ciências e Tecnologia, Universidade Estadual da Paraíba, 58109-790 Campina Grande, PB, Brazil; annapaula_rocha@hotmail.com (A.P.R.d.Q.); raphael.18@hotmail.com (R.L.J.A.); andersoncaetano.s@gmail.com (A.C.S.); jademarinhoqi@hotmail.com (J.M.G.); sueniasoares@yahoo.com.br (S.S.F.); 3 Embrapa Agroindústria de Alimentos, Empresa Brasileira de Pesquisa Agropecuária, 23020-470 Rio de Janeiro, RJ, Brazil; karina.dos-santos@embrapa.br

**Keywords:** by-products upgrading, jabuticaba, probiotics, total phenolic content, instrumental texture, overall acceptability

## Abstract

The use of agro-industrial wastes in combination with indigenous lactic acid bacteria is an interesting option to confer functional potential to food products. The microbial viability, chemical composition, antioxidant capacity, texture and sensory acceptability of a fermented dairy dessert containing the indigenous culture *Lactobacillus plantarum* CNPC003, whey and ingredients obtained from the jabuticaba (*Myrciaria cauliflora*) peel were compared with formulations without lactobacilli (control) or containing a commercial probiotic culture (*Lactobacillus rhamnosus* LR32). *L. plantarum* presented viability higher than 7 log CFU g^−1^ in the dessert, as did the commercial probiotic, for 21 days at 4 ± 1 °C. Total phenolic contents (45–60 mg gallic acid equivalents, GAE, 100 g^−1^) were comparable to those of other studies evaluating dairy products containing plant sources. The formulations were low in fat, presenting as acceptable for overall consumption, with attractive color and appreciable texture. Considering the total antioxidant capacity, 200–250 g of dessert would be necessary to capture 1 g of 1,1-diphenyl-2-picrylhydrazyl (DPPH) radicals. The dessert with *Lactobacillus plantarum* CNPC003 is seen as a viable alternative for the use of whey and jabuticaba peel, as well as a potential functional food due to the concentration of lactobacilli reached, besides the presence of antioxidant phenolic compounds.

## 1. Introduction 

Food products that target improvements in the physiological functions of consumers are known as functional foods [1]. In view of this, scientific studies seek to prove the role of these foods in health promotion and disease prevention, highlighting the role of probiotics as functional food because of their advantages and applications, especially available in dairy products [2,3,4].

Probiotics refer to live microorganisms that, when administered in adequate amounts, confer a health benefit on the host [3]. In parallel, the isolation of new strains of probiotics, which are candidates for the development of functional foods, has been carried out, especially in developing countries, since these microorganisms can promote prevention of chronic diseases and cost reduction with the manufacture and acquisition of dairy products [4,5].

In addition, aiming at reducing costs in the production chain of fermented and unfermented dairy foods, the use of whey and fruits that present high perishability and poor postharvest conservation can contribute to increase the nutritional and functional value of these products [6,7].

The jabuticaba (*Myrciaria cauliflora* Mart*.*) is a tropical fruit native to Brazil, which presents possibility of use in the manufacture of fermented products [8]. Jabuticaba peels are a source of anthocyanins, responsible for the dark color of the fruit. These pigments are powerful antioxidant compounds and present antimicrobial activity [9]. However, jabuticaba peels are generally neglected, which in addition to generating pollutant residues, promotes the waste of nutrients and potentially bioactive compounds present in this portion [10].

Beverages fermented with jabuticaba are traditionally used in folk medicine [8]. Jabuticaba exhibits a high antioxidant capacity to eliminate free radicals and antiproliferative properties in tumor cell lines in vitro [9,11] and, in animal studies, was able to improve the antioxidant potential of blood plasma and control oxidative stress [12,13]. Such characteristics may contribute to the prevention of various diseases, including neurodegenerative disorders [8].

Simultaneous consumption of polyphenols and probiotics may also promote the bioactivation of phenolic compounds in the intestine, increasing the antioxidant activity in the body [13,14]. Likewise, some lactic acid bacteria and microorganisms with probiotic potential may also contribute to the antioxidant potential of food products [14].

Therefore, food products with new probiotic candidates, jabuticaba peels and whey can bring potential benefits to human health, besides reducing environmental impacts by using agro-industrial byproducts. The objective of the present study was to compare the microbial viability, antioxidant capacity, texture and sensory features of a fermented dairy dessert containing whey, ingredients obtained from jabuticaba peel and the indigenous probiotic candidate *Lactobacillus plantarum* CNPC003 with a formulation containing a commercial probiotic culture (*Lactobacillus rhamnosus* LR32) and a control formulation (without lactobacilli adjunct culture).

## 2. Materials and Methods

### 2.1. Obtaining the Jabuticaba Fruits 

The fruits were obtained in the free market of the city of Campina Grande, Paraíba State, Brazil, during the jabuticaba harvest from March to April of 2014 and 2015. They were selected, washed in running water and sanitized with sodium hypochlorite (Neon Comercial, São Paulo, Brazil) diluted in distilled water to obtain a solution of 200 mg L^−1^ free chlorine. Then, the fractions (pulp, peel and seed) were separated by manual pulping and peels were frozen at −18 °C.

### 2.2. Production of the Ingredients Using the Jabuticaba Peels

#### 2.2.1. Syrup and Jam

To reduce the jabuticaba peels astringency through partial hydrolysis and solubilization of the their tannins, this byproduct was treated with distilled water and lemon juice in the proportion 1:2:0.15 (peels:water:lemon juice) for 45 min. The peels were then washed, crushed in a blender with distilled water (90.5 g peels to 170 mL water) and filtered in nylon net (0.300 mm mesh). Sequential crushing was performed with the filtrate and lemon treated peels until obtaining an aqueous extract with 2.5 g 100 g^−1^ soluble solids.

The jabuticaba syrup was produced with the peel aqueous extract (66.39 g 100 g^−1^), sucrose (33.19 g 100 g^−1^) and pectin (Grindsted^®^ Pectin YF 310, Danisco Mexicana, DuPont, Apatzingán de la Constitución, Mexico, 0.42 g 100 g^−1^), with heating to achieve 40 g 100 g^−1^ soluble solids.

The jam was produced by heating the aqueous extract (66.412 g 100 g^−1^) with sucrose (33.174 g 100 g^−1^), pectin (Grindsted^®^ Pectin YF 310, Danisco Mexicana, DuPont, Apatzingán de la Constitución, Mexico, 0.41 g 100g^−1^) and cochineal carmine dye (ILCASA, Indústria de Laticínios de Campina Grande S/A, Campina Grande, Brazil, 0.004 g 100g^−1^) until this mixture was concentrated to a content of 60 g 100 g^−1^ soluble solids.

#### 2.2.2. Hydroethanolic Extract

The hydroethanolic extract was obtained from the solid residue after filtration of the aqueous extract, using the method adapted from dos Santos et al. [14] for the use of waste from the wine industry with modifications. Initially, 10 g of the filtrate solid residue was hydrated with 5 mL of distilled water for 1 h. Then, 90 mL of hydroethanolic solution was added to the hydrated residue, prepared in the proportion of 30 mL of extra neutral ethanol (Usina Giasa, Biosev, Pedras de Fogo, Brazil) to 100 mL of solution, which was acidified with citric acid (Arcólor, São Paulo, Brazil) up to pH 4 to facilitate the extraction of the phenolic compounds. Next, the residue in hydroethanolic solution was sonicated in an ultrasonic bath (50 rpm) for 2 h at 50 °C. Finally, the hydroethanolic extract was filtered in a nylon net (0.300 mm mesh) and allowed to dry in an air circulation oven (Nova Ética, Vargem Grande Paulista, 50 °C) to evaporate the ethanol and concentrate to 5–6% of the initial volume. The hydroethanolic extract was stored in cryogenic tubes (Kasvi, São José dos Pinhais, Brazil) at −18 °C. 

### 2.3. Processing of Cheese to Obtain Whey

Bovine milk whey was obtained from the rennet-coagulated cheese processing described by Florentino [15], with some modifications, using skimmed pasteurized milk (Cariri Light, Cooperativa Agropecuária do Cariri Campina Grande, Brazil), Hannilase coagulant (Chr. Hansen, Valinhos, Brazil), added according to the manufacturer’s instructions, and calcium chloride (Neon Comercial, São Paulo, Brazil, 0.25 g L^−1^ milk). The milk was first heated to 34–37 °C for addition of the other ingredients, with stirring. The milk was kept at rest for approximately 45 min until the coagulation was complete, when the curd was cut. After subsequent periods of stirring and resting of the curd, whey drainage and cheese shaping were performed. The whey was packed in nylon plastic bags and stored at −18 ° C until the moment of its use.

### 2.4. Recovery of the Potentially Probiotic Indigenous Culture of Lactobacillus plantarum CNPC 003

*Lactobacillus plantarum* strain CNPC003 (formerly *L. plantarum* B12) was isolated from goat milk derivatives and previously evaluated for its probiotic potential by Embrapa Goats and Sheep, Sobral, Ceará State, Brazil [5]. The indigenous strain of *L. plantarum* CNPC003 was supplied by Embrapa Goats and Sheep in the lyophilized form. An arbitrary aliquot of the lyophilized culture powder was grown in 10 mL of de Man Rogosa Sharpe (MRS) broth (Difco, Sparks, MD, USA) for 24 h at 37 °C. The broth was distributed in 1.5 mL microtubes and bacterial culture was harvested by centrifugation with discarding of the supernatant. After this procedure, the culture was washed three times with sterile saline solution (NaCl, Neon Comercial, São Paulo, Brazil, 0.85 g 100 mL^−1^) for incorporation into the dairy base to be fermented.

### 2.5. Production of Fermented Dairy Desserts

Three pilot-scale trials of fermented dairy dessert, denoted T1, T2 and T3, were carried out in three batches (genuine replicates), all of them added with hydroethanolic extract and syrup produced from jabuticaba peels, accompanied by jam also produced from the same byproduct: T1 (control), with the starter culture of *Streptococcus thermophilus* TA40 (Danisco DuPont, Dangé, France); T2 (commercial probiotic), with *S. thermophilus* TA40 and the potentially probiotic commercial culture of *L. rhamnosus* LR32 (Danisco DuPont, Madison, WI, USA); and T3 (experimental), produced with *S. thermophilus* TA40 and the indigenous *L. plantarum* CNPC003 culture. 

First, the whey was thermally pre-treated at 85 °C for 5 min for inactivation of the coagulant enzymes. The dairy base was obtained from the mixture of pre-treated whey (840 g kg^−1^) with sucrose (80 g kg^−1^) and skim milk powder (Molico*,* Nestlé, Araçatuba, Brazil, 80 g kg^−1^), and heat treated at 85 °C for 30 min.

When the dairy bases reached 40 °C, cultures *S. thermophilus* TA40 (0.03 g kg^−1^ in the three trials), *L. rhamnosus* LR32 (0.2 g kg^−1^ in the T2 trial) and *L. plantarum* CNPC003 (after activation in T3 trial) were added. The dairy bases were kept in an oven at 43 ± 2 °C until a pH of less than or equal to 5.0 was obtained. After fermentation, the dairy bases (900 g kg^−1^) of each separate treatment were mixed in a blender together with jabuticaba peel syrup (57.655 g kg^−1^), hydroethanolic extract of jabuticaba peel (20 g kg^−1^), pectin (Grindsted® Pectin YF 310, Danisco Mexicana, DuPont, Apatzingán de la Constitución, Mexico, 17.5 g kg^−1^), lactic acid (FCC85 food grade solution at 85 g 100 g^−1^, Purac, São Paulo, Brazil, 4.8 g kg^−1^ final desserts) and cochineal carmine dye (0.450 g kg^−1^). Portions of 75 g of the resulting dairy desserts were packed in individual plastic pots with lid (high density polypropylene), pre-added with 12–13 g jabuticaba peel jam. Twelve pots of each treatment were filled for each batch produced. The list of the ingredients used to produce the fermented dairy desserts is shown in Table 1.

### 2.6. Storage and Sampling Periods

Fermented dairy desserts were stored under refrigeration at 4 ± 1 °C and sampled after 1, 7, 14 and 21 days. For all trials, samples of each batch in each sampling period were frozen at −18 °C for the determination of the total phenolic contents. Two batches of each trial were also used for the evaluation of antioxidant capacity. The mean composition was evaluated for the fermented milk desserts of the three batches of the three treatments, from frozen samples on the first day of storage. The milk bases during the fermentation process were also sampled for pH and microbiological parameters.

### 2.7. Determination of the Mean Composition of Fermented Dairy Desserts

The total solids content was obtained by drying 2 g of sample in a vacuum oven Marconi (model MA 030/12, Piracicaba, Brazil) at 70 °C [16]. The ash content was determined by the incineration of 2 g of sample at 550 °C until the total elimination of organic matter [16]. The fat content was obtained by solvent extraction using the method of Folch et al. [17]. The protein content was estimated from the analysis of the nitrogen content by the micro Kjeldahl method, using the conversion factor of 6.38 for milk and dairy products [18]. The total carbohydrate content was calculated by difference to obtain 100% of the total composition [19]. All parameters were obtained in triplicate.

### 2.8. Physicochemical Parameters and Viability of Starter Cultures and Adjuvants

The pH values obtained in a TEC-5 pH-meter (Tecnal®, Piracicaba, Brazil) were determined in the milk bases immediately after the addition of the cultures (initial time = 0 h) and during fermentation at 1 h intervals, as well as in the desserts after 1, 7, 14 and 21 days of storage, in duplicate, according to the analytical procedures of the Instituto Adolfo Lutz [16].

The titratable acidity, expressed in grams of lactic acid 100 g^−1^, was determined in duplicate, according to the analytical standards of the Instituto Adolfo Lutz [16], in desserts after 1, 7, 14 and 21 days of storage.

The microbiological analyses were carried out in triplicates on the dairy bases before and after the fermentation, aseptically transferring 1.0 mL of the sample to 9.0 mL of saline solution (NaCl, Neon Comercial, São Paulo, Brazil, 0.85 g 100 mL^−1^), as well as in the desserts along the storage, with the dilution of 25 g of each dessert in 225 g of saline solution. Decimal dilutions of each sample were obtained using the same diluent. The viability of *S. thermophilus* in dairy bases and desserts was determined by pour plating 1 mL of each dilution in M17 agar (Difco, Sparks, MD, USA), with added lactose (Vetec, Sigma Aldrich, Duque de Caxias, Brazil, 5 g L^−1^), followed by incubation at 37 °C for 48 h [14,20]. The viability of *Lactobacillus* sp*.* in the milk bases and desserts of the T2 and T3 treatments was determined by pour plating 1 mL of each dilution in MRS agar (Difco, Sparks, MD, USA) acidified to pH 5.4 with acetic acid (Vetec Química Fina, Duque de Caxias, Brazil), followed by incubation at 37 °C [14] for 48 h.

### 2.9. Extraction of Phenolics for Analysis of Total Phenolic Content and Antioxidant Capacity

The extracts of the fermented milk dessert samples were obtained according to dos Santos et al. [14], with some modifications. Samples of desserts (1.25 g) were mixed with 5 mL of methanol–HCl (concentrated HCl, Qhemis, Jundiaí, Brazil, in methanol, Qhemis, Jundiaí, Brazil, 0.1 mL 100 mL^−1^), and left overnight at 4 °C. The cooled mixtures were then centrifuged (centrifuge 5810R, Eppendorf, Hamburg, Germany) at 13,500 × *g* for 5 min at 4 °C. The residue was washed with methanol–HCl, repeating the procedure four times. The supernatants obtained were used for the analyses.

### 2.10. Total Phenolic Analysis

The total phenolic content was determined according to dos Santos et al. [14] with some modifications. All procedures were carried out in the dark. Aliquots of 60 μL of each prepared extract, 2340 μL of distilled water and 150 μL of Folin–Ciocalteau reagent (Sigma-Aldrich Chemie GmbH, Steinheim, Germany) were transferred into test tubes and mixed. After 8 min, 450 μL of Na_2_CO_3_ solution (Na_2_CO_3_, Neon Comercial, São Paulo, Brazil, 30 g 100 mL^−1^) were added to the tubes, again mixed, and left at rest for 30 min at room temperature. The absorbance was measured at 750 nm in a SP-2000 UV spectrophotometer (Spectrum, Shanghai, China) and a standard curve was constructed using gallic acid (Vetec, Sigma-Aldrich, Duque de Caxias, Brazil). The results were expressed as mg gallic acid equivalent (mg GAE) 100 g^−1^ sample. The samples were analyzed in triplicates.

### 2.11. DPPH Assay and Antioxidant Capacity Calculation

The antioxidant capacity of dessert trials by 1,1-diphenyl-2-picrylhydrazyl (DPPH) radical scavenging assay was determined according to the method proposed by Karaaslan et al. [21] with modifications. Different aliquots of sample extracts (50 μL, 100 μL and 200 μL) were mixed with aliquots of 100 μM DPPH (2.95 mL, 2.90 mL and 2.80 mL, respectively) for a total volume of 3 mL. The decrease in absorbance at 517 nm was measured after 60 min of sample maintenance at room temperature.

The results obtained were expressed as percentage of DPPH inhibition (% DPPH Scavenging Effect), following equation (1):

(1) $$\mathrm{DPPH}\;\mathrm{Scavenging}\;\mathrm{Effect}\;(\%)=\frac{(\mathrm{Ac}-\mathrm{As})\;}{\mathrm{Ac}}\times 100$$

where Ac is the absorbance of the control (absorbance of the DPPH solution without the sample extract) and As is the absorbance with the sample extract.

The amount of dessert required to reduce the initial concentration of DPPH by 50% (EC_50_) was calculated (initially in g of sample per L of 100 μM DPPH solution) after constructing the percent inhibition by the concentration curve of the extract. The final result of total antioxidant capacity was expressed in g of DPPH g^−1^ sample, according to Rufino et al. [22] following Equation (2):

(2) $${\;\mathrm{Total}\;\mathrm{antioxidant}\;\mathrm{capacity}\;(g\;\mathrm{sample}\;g\;}^{-1}\;\mathrm{DPPH})=\frac{{\mathrm{EC}}_{50}{\;(g\;L}^{-1})}{\mu M\;\mathrm{DPPH}\times 394.3}\times 10^{6}$$

where μM DPPH is the DPPH (in μM) consumed by the dessert to decrease the absorbance by 50% during the assay and 394.3 is the DPPH molar mass.

### 2.12. Instrumental Determination of Texture of Fermented Dairy Desserts

Texture was instrumentally determined using the back extrusion test in duplicate samples of each batch immediately withdrawn from the storage condition (4 ± 1 °C) with a TA.XT Plus Texture Analyzer (Stable Micro Systems, Surrey, UK). A compression test was performed on 87-88 g samples in the individual plastic pots by a 35 mm diameter acrylic disc (Stable Micro Systems, Surrey, UK). At the beginning of each test, the acrylic disk was set at 20 mm above the top of sample surface. Penetration distance, penetration speed and return speed of 30 mm, 1 mm s^−1^ and 10 mm s^−1^, respectively, were used. Firmness, consistency, cohesiveness, and viscosity index were determined using the “Exponent” software (version 6.1.4.0, Stable Micro Systems).

### 2.13. Sensory Evaluation of Fermented Dairy Desserts

The sensory evaluation of this study was approved by the Ethics Committee of the State University of Paraíba (Certificates of Presentation for Ethical Appreciation (CAAE), No. 43582514.3.0000.5187 and No. 43591315.2.0000.5187) and was conducted at the Laboratory of Sensory Analysis of the Academic Unit of Food Engineering of the Federal University of Campina Grande, Paraíba State, Brazil. The analyses of fermented dairy desserts were performed after 7 and 21 days of storage (4 ± 1 °C) through acceptability test, using the 11-point hybrid hedonic scale ranging from 10 (“liked extremely”) to 0 (“disliked extremely”) [23]. The evaluation was made by 35 untrained judges in each session, previously selected based on interest and fruit-added dairy products consuming habits. This audience was composed of healthy adults, mainly students and staff (18 males and 17 females in the first session; 23 males and 12 females in the second session; one session for each storage time). Transparent and individual plastic cups containing 12 g of product were coded with three random digits. Samples stored at 4 ± 1 °C were maintained at this temperature before being served. Three samples, one of each trial, were served, being delivered at the same time to each judge, who was oriented to experiment from left to right, observing the randomization to avoid the “order effect” [24]. The judges were also instructed to report attributes related to taste, texture, appearance and color of the products which were “most appreciated” and “less appreciated” in the samples analyzed. The judges were free to mention more than one attribute or none at all as they wished. For the fermented dairy desserts were previously analyzed on the first day of storage for microbiological sanitary parameters (total coliforms, thermotolerant coliforms and *Salmonella* sp.), thus promoting the safety of the judges.

### 2.14. Statistical Analysis

Data were presented as mean ± standard deviation. Initially, the data were analyzed for normality using the Shapiro–Wilk test and homogeneity of variances using the Bartlett test. When these assumptions were confirmed, the data were submitted to analysis of variance and the means were compared by the Tukey test, with a level of 5% of significance. The other data were analyzed by means of nonparametric equivalent tests. Statistical analyses were performed using the Statistica 8.0 program (Statsoft Inc., Tulsa, OK, USA).

## 3. Results

### 3.1. Mean Composition of Fermented Dairy Desserts

The results of the mean composition of probiotic fermented dairy desserts on the first day of storage under refrigeration at 4 ± 1 °C are shown in Table 2. The addition of the adjunct cultures (commercial probiotic *L. rhamnosus* LR32 and indigenous *L. plantarum* CNPC003 in trials T2 and T3, respectively) did not interfere in the nutritional composition of fermented dairy desserts, since there was no significant difference (*p* > 0.05) between the dessert trials (T1, T2 and T3), regarding the different parameters analyzed (total solids, fat, ash, protein and total carbohydrates). The total solids content of the fermented dairy desserts was mainly composed of total carbohydrates. Regarding the fat and protein contents, all dairy desserts obtained values lower than 0.5 g 100 g^−1^ and higher than 2 g 100 g^−1^ of product (fresh weight sample), respectively.

### 3.2. pH Values and Viability of Starter and Adjuvant Cultures in the Dairy Bases during the Fermentation Process

**Figure 1:**
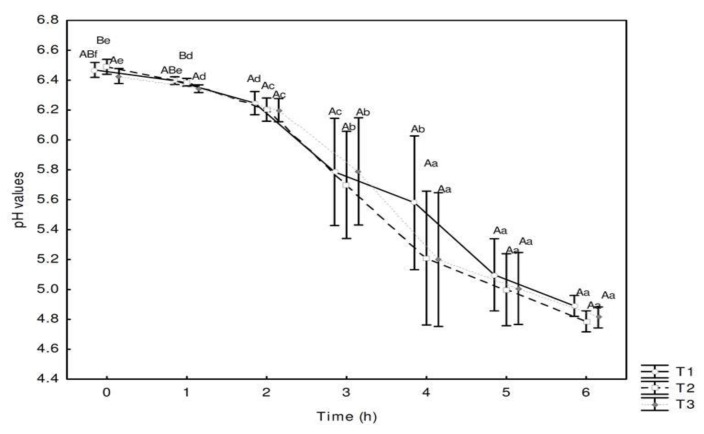
Changes in the mean values of pH of dairy bases during the fermentation of T1 (control, without lactobacilli adjunct), T2 (commercial probiotic, with *L. rhamnosus* LR32) and T3 (experimental probiotic, with the indigenous culture *L. plantarum* CNPC003) trials. Different uppercase letters denote significant differences between trials for the same fermentation time (*p* < 0.05). Different lowercase letters denote significant differences between fermentation times for the same trial (*p* < 0.05).

The pH values obtained during fermentation of the fermented dairy base of the T1, T2 and T3 trials is shown in Figure 1.

During the fermentation process, pH values differed between trials for the same fermentation sampling period only at the initial times (0 h and 1 h), when T3 (with *L. plantarum* CNPC003) had significantly lower values (*p* < 0.05) than T2 (with *L. rhamnosus* LR32). Regarding the fermentation time, there was a successive significant pH reduction within the same trial between 0 h and 4 h (*p* < 0.05) and pH stability, without significant differences for up to 6 h for treatments T2 and T3. Such behavior did not occur with the T1 control dessert (without adjuvant culture), which showed successive pH reduction for up to 5 h of fermentation. On the other hand, from the fifth hour, the mean values of pH were very close to the three trials within the same batches. After the end of the fermentation process, the mean pH values for trials T1, T2 and T3 were 4.89 ± 0.16, 4.79 ± 0.04, and 4.81 ± 0.06, respectively.

**Figure 2:**
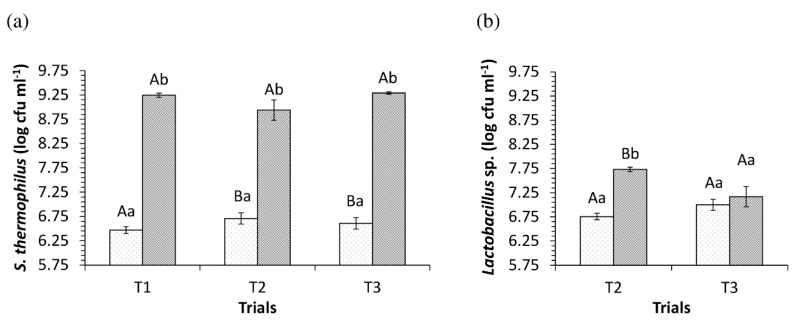
Populations of *S. thermophilus* in T1 (control), T2 (*L. rhamnosus* LR32) and T3 (*L. plantarum* CNPC003) trials (**a**) and of *Lactobacillus* sp*.* in T2 and T3 trials (**b**) during the fermentation of the dairy bases (light grey dots = initial time; grey square angle = final time). Different uppercase letters denote significant differences between trials for the same fermentation time and microorganism (*p* < 0.05). Different lowercase letters denote significant differences between fermentation times for the same trial and microorganism (*p* < 0.05).

The populations of *S. thermophilus* and *Lactobacillus* sp*.* in the dairy base trials T1, T2 and T3 in the initial and final times of the fermentation process are shown in Figure 2.

*S. thermophilus* populations at the beginning of fermentation were significantly lower in T1 (*p* < 0.05); however, T2 and T3 did not differ among them for this microorganism at the initial time (*p* > 0.05). On the other hand, *S. thermophilus* values increased significantly by more than two log cycles between the initial and final fermentation times in the three trials (*p* < 0.05) without resulting in significant differences between the three trials at the end of fermentation (*p* > 0.05). 

In relation to the populations of *Lactobacillus* sp*.*, no differences were observed between trials T2 and T3 at the beginning of fermentation (*p* > 0.05); however, these trials differed significantly among themselves at the end of the incubation process at 43 ± 2 °C (*p* < 0.05), with higher populations in the product containing *L. rhamnosus* (T2), although pH values were observed without difference at the end of the fermentation (*p* > 0.05). Considering the isolated behavior of *Lactobacillus* sp*.* in each of the desserts (T2 and T3), a significant increase was observed only in that containing *L. rhamnosus* (*p* < 0.05).

### 3.3. Physicochemical Parameters and Viability of Starter and Adjuvant Cultures during the Storage of the Dairy Desserts with the Ingredients of Jabuticaba Peel

The pH, titratable acidity and populations of *S. thermophilus* and *Lactobacillus* sp*.* during the storage days (1, 7, 14 and 21) of the fermented dairy desserts is shown in Table 3.

The final products (Table 3) presented lower pH values than those observed at the end of the fermentation of the milk bases (Figure 1) due to the ingredients derived from the jabuticaba peel and the lactic acid added. However, there was no significant difference (*p* > 0.05) between the three dessert trials in relation to pH in the storage period, showing that the use of the adjuvant cultures studied, especially the indigenous *L. plantarum* CNPC003 culture in the T3 trial, resulted in a stable pH in the products under refrigerated conditions, without post-acidification, similar to that occurred for the T1 control dessert, containing only the starter culture of *S. thermophilus.*

On the other hand, the ingredients added to the final product did not interfere in the populations of the starter and adjuvant microorganisms on the first day of storage (Table 3), since they were close to those observed at the end of the fermentation of the milk bases (Figure 2).

The populations of *S. thermophilus* did not differ among the three trials within each sampling period (*p* > 0.05), indicating that the adjuvant cultures used, particularly the indigenous culture (*L. plantarum* CNPC003), did not interfere in the viability of the starter microorganism during the storage. However, a significant reduction was observed for all trials between the first and seventh days in the *S. thermophilus* population (*p* < 0.05). Populations of the starter microorganism were higher than 9 log CFU g^−1^ at the beginning of storage and decreased below this value from the second week, but close to or greater than 8.90 log CFU g^−1^ by the 21st day.

Regarding the viability of *Lactobacillus* sp*.*, comparing the T2 products with T3, there was a significant difference between these treatments up to 14 days of storage (*p* < 0.05), with the population of the adjuvant culture in T2 being higher in at least 0.5 log cycle compared to T3 due to the population of *L. rhamnosus* LR32 having been higher in the dairy base at the end of the fermentation (Figure 2). A significant reduction in the populations of *Lactobacillus* sp*.* was also verified in both trials from the fourteenth day of storage compared to the first day (*p* < 0.05).

### 3.4. Total Phenolic Compounds and Antioxidant Capacity of Dairy Desserts with the Ingredients of Jabuticaba Peel

The mean values of phenolic compounds (in mg of GAE 100 g^−1^), as well as the percentage of inhibition of DPPH radicals, the EC_50_ and the antioxidant capacity of the sample (in g of sample g^−1^ of DPPH) for T1, T2 and T3 trials over of the storage period at 4 ± 1 °C are shown in Table 4.

There was no significant difference (*p* > 0.05) between the trials studied in relation to the content of phenolic compounds and antioxidant capacity in the storage period (1, 7, 14 and 21 days), which may be indicative of good interaction between the probiotic microorganisms, total phenolics and other components of the desserts. It is worth mentioning that the product with the indigenous adjuvant culture of *L. plantarum* CNPC003 presented characteristics similar to the control product containing the starter culture and the commercially available adjuvant probiotic culture *L. rhamnosus*.

In relation to the percentage of inhibition of DPPH radicals (%), the average values obtained in the present study for all dairy desserts containing products derived from jabuticaba peel, at the maximum aliquot used in the assay (0.02 mL of sample extract for a total volume of 3 mL with 100 μM DPPH), were greater than 30% inhibition of DPPH radicals. The EC_50_ values in g of L^−1^ sample of 100 μM DPPH solution from this study were less than 7 g L^−1^.

Considering the final results of the total antioxidant capacity of the samples of the present study, 200–250 g of dessert would be necessary to capture 1 g of DPPH radicals, which is feasible to be consumed at each eating occasion.

### 3.5. Instrumental Texture of Fermented Dairy Desserts

The instrumental texture parameters of the fermented dairy desserts are presented in Table 5. The three dairy dessert trials (T1, T2 and T3) did not differ among them in relation to firmness and consistency parameters in the same storage period (*p* > 0.05). However, on the first day of storage, the T2 formulation with the commercial *L. rhamnosus* culture had a significantly lower (*p* < 0.05) cohesiveness value, whereas the T3 formulation with the indigenous *L. plantarum* culture had a significantly higher viscosity index (*p* < 0.05). However, significant differences between treatments were no longer verified for these parameters after 14 days of storage.

On the other hand, significant differences were observed between the sampling periods (*p* < 0.05) for each trial was assessed separately throughout the storage. For the T1 trial, there was a significant increase of the four instrumental texture parameters during the first week of storage (*p* < 0.05) and a significant reduction at 14 days (*p* < 0.05), except for the viscosity index. For the T2 trial, only the cohesiveness increased significantly in the first week, and the consistency of this dessert reduced significantly between 14 and 21 days (*p* < 0.05). For T3, significant increases in consistency and cohesiveness were observed in the first week of storage (*p* < 0.05), while a significant reduction of consistency at 21 days compared to Day 7 and of the viscosity index from 14 days was also observed (*p* < 0.05).

### 3.6. Sensory Evaluation of Fermented Dairy Desserts

**Figure 3:**
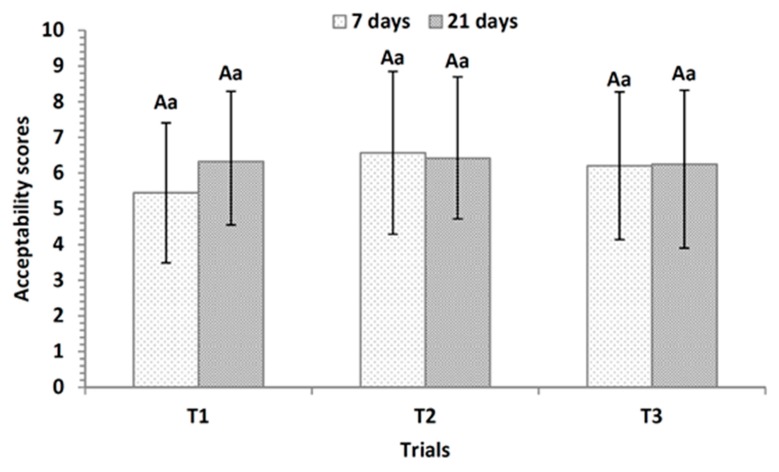
Overall acceptability values (average ± standard deviation) obtained in the sensory analysis with judges (*n* = 35) for the fermented dairy dessert trials containing jabuticaba peel ingredients after 7 days and 21 days storage. T1 = control, without lactobacilli adjunct; T2 = commercial probiotic, with *L. rhamnosus* LR32; T3 = experimental probiotic, with the indigenous culture of *L. plantarum* CNPC003. ^A^ = Same uppercase letters do not differ significantly between the trials studied (*p* > 0.05). ^a^ = Same lowercase letters do not differ significantly over time for the same trial (*p* > 0.05).

Overall acceptability in the present study (Figure 3) did not differ significantly between the desserts evaluated on any day of storage, and no significant differences were observed for the same trial throughout storage (*p* > 0.05).

In the present study, it can be observed that the different cultures employed in the desserts did not interfere with the overall acceptability of the judges and they did not result in significant changes that could be perceived by means of sensory evaluation over time. Considering that most of the average values were between 6.0 and 7.0, they can be interpreted as “slightly like”. For the three fermented dairy desserts trials, texture and color together received about 60% of the total citations as the “most appreciated” attributes by the judges on both Day 7 and Day 21 (Table 6). Flavor received, in general, 20–30% of the citations as the “most appreciated” and 50–60% as “less appreciated” (Table 6), particularly due to the predominant acidic taste of the formulations, as highlighted by the judges.

## 4. Discussion

The dairy desserts of this study are important matrices with low fat and carriers of dairy proteins. The main ingredients of the fermented dairy desserts of this study were skimmed milk powder, whey, sugar added to the milk base for fermentation, jabuticaba peel syrup and jam, and pectin, which did not provide considerable amount of fat to the formulations. The average fat content of the samples was less than 0.5 g 100 g^−1^, which could classify the samples as “fat-free” according to the claims relating to nutrient content of the foods proposed by the Codex Alimentarius [25]. Regarding the Brazilian legislation, the three trials could be classified as “low in total fat” products since the limit, according to the Brazilian Health Regulatory Agency, is 3.0 g of total fat in a serving size of the product [26] being 120 g for dairy desserts [27]. In view of this, the claim of “low-fat dessert” may be considered an attractive characteristic for the consumption of indulgence products with probiotic properties. Moreover, milk proteins are recognized by their high nutritional value due to the presence of all essential amino acids [28]. Protein values of the desserts studied remained higher than 2.0 g 100 g^−1^ of edible portion, being consistent with the values obtained for other whey-enriched dairy products, such as the dairy beverages studied by Rufino et al. [29] with a protein content of 2.30 g 100 g^−1^. 

Regarding the behavior of the adjuvant lactobacilli during the fermentation process, the results of this study were similar to those of Buriti et al. [20] which highlighted the stimulation of *L. rhamnosus* LR32 commercial culture during the incubation step (at 43 ± 2 °C) of dairy bases containing goat milk, sugar and goat cheese whey, in which the population of this microorganism showed a significant increase in this step, from 6.91 log CFU mL^−1^ to 7.13 log CFU mL^−1^ (*p* < 0.05), and also during the cooling process (*p* < 0.05), reaching values of 8.11 log CFU mL^−1^. On the other hand, although adjuvant strains with probiotic potential are described as capable of having their proliferation enhanced when grown in co-cultures during milk fermentation [30], the indigenous culture of *L. plantarum* CNPC003 was not stimulated in the fermentation conditions employed in this study (43 ± 2 °C) when co-cultured with the starter *S. thermophilus* TA40 in the presence of substrates added to the milk base (milk powder, whey and sugar), as opposed to that observed for the commercial adjuvant *L. rhamnosus*. Georgieva et al. [31] reported that *L. plantarum* may show limited growth capacity in milk due to its weak proteolytic activity, which might explain the lack of increase in the population of indigenous *L. plantarum* CNPC003 strain in the T3 dairy bases during fermentation. Likewise, Settachaimongkon et al. [32] observed that populations of *L. plantarum* WCSF1, precultured or not under stress conditions at different pH values (4.5 or 6.5) and NaCl concentrations (1.5% and 4.5%), although viable, remained unchanged during the fermentation of yoghurt when co-cultured with *S. thermophilus* C44 and *Lactobacillus delbrueckii* subsp*. bulgaricus* C49.

In relation to the behavior of the starter culture in the final product, the viability of *S. thermophilus* TA40 observed in the study was not influenced by the lactobacilli adjuncts. These results were consistent with Pereira et al. [33] that found *S. thermophilus* TA 40 values near 8 log CFU g^−1^ during 28 days of storage at 4 ± 1 °C for petit Suisse cheeses produced with a co-culture of the probiotics *Lactobacillus acidophilus* LA C4 and *Bifidobacterium animalis subsp. lactis* DN 173-010, in addition to the supercritical extract of the jabuticaba peel at the concentration of 5000 mg kg^−1^ of final product.

Other parameters such as the titratable acidity and pH are also usually used to determine the milk quality before and during the production of fermented milk products [34], which are highly dependent of the starter micro-organism used and its interaction with the adjuvant cultures [35]. Considering the effects of the lactic cultures on the titratable acidity of products, the results for desserts of this study were consistent with the values typically observed for other fermented dairy products marketed in Brazil, such as fermented milks (0.6–2.0 g of lactic acid 100 g^−1^) and yoghurts (0.6–1.5 g of lactic acid 100 g^−1^), as recommended by the Brazilian regulatory standards [36]. Moreover, these results were not influenced by the lactobacilli adjuncts. Regarding the pH values, Sidira et al. [37], when evaluating the use of free and immobilized cells of *L. plantarum* 2035 strain isolated from Greek feta cheese for the production of probiotic yogurts with *Lactobacillus delbrueckii* subsp*. bulgaricus* and *S. thermophilus* from the CH1 commercial culture, observed that, during storage at 4 °C (1, 8, 15, 22 and 29 days), the pH in the first days decreased, but remained stable in all samples of yoghurts, without signs of post-acidification, similar to what occurred for the desserts in the present study. Sidira et al. [37] also observed that populations of *S. thermophillus* remained stable throughout storage whereas, on the contrary, those of *Lactobacillus* spp. decreased. However, the authors confirmed by multiplex polymerase chain reaction (PCR) that *L. plantarum* 2035 was able to maintain above 6 log CFU g^−1^, considered adequate to confer probiotic effect according to the requirement of the commercial sector in Greece. Considering the probiotic potential of the adjuvant cultures used in the desserts of the present study, the viability of *L. rhamnosus* and *L. plantarum*, in treatments T2 and T3, respectively, was within what is recommended by the scientific literature, being active and viable above 10^6^ CFU g^−1^ throughout the storage or between 10^8^–10^9^ CFU g^−1^ within the 100–120 g serving portion of this type of product customarily consumed per eating occasion [38]. 

Fruits, fruit extracts and other plant food ingredients are important contributors to the content of phenolic compounds in different dairy products. Moreover, some lactic acid bacteria can metabolize such compounds in specific conditions, particularly *L. plantarum* [39]. This characteristic was not verified in the present study since the total phenolic content of the desserts of the present study remained similar for all trials during storage and were comparable to those obtained in a previous study of this research group for fermented milks with *S. thermophilus* TA40 in co-culture with *L. acidophilus* LA-5 or *L. rhamnosus* HN001 added with grape juice and grape pomace extract, which remained close to 45 mg GAE 100 g^−1^ for 28 days of storage [14].

In the context of antioxidant capacity, desserts with either *L. rhamnosus* LR32 or *L. plantarum* CNPC003 showed similar behavior to that verified for the control product during the storage. However, the comparison of these results with products from different studies requires some care due to the use of different dilutions of the samples for the analysis, different types of solvents and methods to obtain the results. Each sample has antioxidant power and behaves differently in each type of analysis [40]. Regarding the percent inhibition of DPPH radicals, the results found in this study were higher than those found by Shori [41] for plain-cow-milk yogurt during 21 days of storage, which had between 20% and 30% inhibition of DPPH radicals. On the other hand, values of up to 60% inhibition of DPPH radicals were obtained by that author for yogurt with a mixture of cow milk and soybean water-soluble extract. Furthermore, in the context of EC_50_ values from this study (g of sample L^−1^ of 100 μM DPPH solution), the results were much lower than those observed by Padua et al. [40] when evaluating a banana (*Musa* AAB, Prata subgroup) flavored yoghurt enriched with jabuticaba peel meal (342.19 g L^−1^). These values indicate a superior antioxidant activity of the desserts of the present study, since the lower the value of EC_50_ the greater the antioxidant activity. On the other hand, Caleja et al. [42] in the evaluation of fortified yogurts with natural additives based on fennel (*Foeniculum vulgare* Mill.) and chamomile (*Matricaria recutita* L.) decoctions, concluded that the yogurts incorporated with chamomile expressed EC_50_ values of 16.4 ± 0.8 mg mL^−1^ and 45% inhibition of DPPH, presenting similarities to the findings for the dessert trials analyzed in the present study.

In the development of functional foods, the study of their texture parameters and sensory acceptability also has received important attention in parallel to the investigation of their potential health benefits [20,37,40,43]. This is of particular concern when probiotic adjuncts are added to dairy products since their texture can be affected by these cultures [35], as verified for the desserts of the present study during storage. According to Szczesniak [44], “texture is the sensory and functional manifestation of the structural, mechanical and surface properties of foods detected through the senses of vision, hearing, touch and kinesthetic”. The texturometer is one of the possibilities to evaluate the texture of foods, through a device similar to the human senses [44,45]. The TA.XT Plus Texture Analyzer, used in the present study, is basically able to measure any physical property of the product, which continuously records the force, distance at a time, simultaneously while deformation of the material with pressure or tensile force [45] 

For Szczesniak [44], firmness is an example of a mechanical sensory parameter that is relatively straight forward with a texturometer since it is on a scale of resistance of food to the applied compressive forces. Complementarily, the physical definition of cohesiveness is the extent to which a material can be deformed before it ruptures, while viscosity is considered the rate of flow per unit of force [44]. In addition, a relative measure of consistency of a product is the amount of “work to extrude”, which can be found by determining the positive area under the force-distance curve when using a back extrusion test [46], as performed in this study.

Similar to that observed in the present study, Costa et al. [43] when evaluating instrumental parameters in goat's milk yogurt containing only probiotics or probiotics with cupuaçu (*Theobroma grandiflorum*) pulp over 28 days of storage, showed a trend of loss of the texture of the product throughout the storage, being significant for apparent viscosity and firmness (*p* < 0.05), although significant for consistency (*p* < 0.05) for the yoghurt with only probiotic. For the other formulations of goat milk yogurts evaluated in that study (natural, prebiotic natural, synbiotic natural and cupuaçu without probiotic), only apparent viscosity significantly reduced throughout storage (*p* < 0.05). According to the authors, such behavior is highly dependent on the composition of the microbial cultures used in the fermentation, total solids and protein content of the product. Thus, the different co-cultures used in the preparation of the desserts could justify, in part, the texture variations obtained in the present study. In addition, considering that the desserts studied were low in fat content, the highly reduced amount of this nutrient could also explain the decrease in consistency from the second half of the storage period. The fat globules interact with casein micelles and participate in the formation of the protein gel, collaborating both for the texture of the products and for their stability during storage. According to Guggisberg et al. [47] based on results from confocal laser scanning microscopy (CLSM), fat, when present in fermented dairy products, allows the formation of a gel with closed microstructure connected by fine lines and well-defined pores, whereas products with very low fat content (ca. 0.2 g 100 g^−1^) result in a less compact protein network. This characteristic, therefore, would make low fat products, such as those of the present study, more susceptible to loss of consistency over time.

In the development of dairy products with probiotics, there is another great concern that such microorganisms do not result in significant sensory changes compared to similar products without probiotics as adjunct cultures or during storage time [35]. In the present study, the changes observed for the texture determined instrumentally in the desserts during storage did not interfere in the acceptability of these products. Some studies show that the sensory characteristics of dairy products with probiotic lactobacilli is mainly affected by their acidity [48,49,50]. In the study by Buriti et al. [48], it was found by preference test that fresh cream cheese with the addition of *Lactobacillus paracasei* LBC82 in co-culture with *S. thermophilus* TA40 showed a more acidic taste and was significantly less preferred over the control, with only *S thermophilus.* Bayarri et al. [49], when evaluating the acceptability of commercial probiotic plain yogurts and plain fermented milks, all of them semi-skimmed or skimmed, using a nine-point hedonic scale with 120 consumers, obtained mean values close to 5.0 for two products containing *Bifidobacterium* and equal to 6.0 for the product containing *Lactobacillus casei*. The highest averages obtained by these authors were for yogurts without probiotics (between 6.5 and 7.5). In turn, the study by Dias et al. [50] when evaluating the acceptability values of a synbiotic fermented dairy beverage with addition of *L. acidophilus* LA-5 using the nine-point hedonic scale, showed changes throughout storage, although considered good, since between zero and seven days of storage the mean scores were close to 5.0 (equivalent to “neither like nor dislike”), which increased significantly between 14 and 21 days (*p* < 0.05), reaching values close to 6.0 (equivalent to “slightly like”), also in attribution to the increase of the acidity.

The acidic taste, which was rated as less appreciated by the judges in the studied samples, demonstrates that this characteristic remained similar for all products evaluated in both sampling periods of sensory analysis. Since the pH values and titratable acidity of the desserts did not vary between treatments and throughout storage (Table 2), the perceived acidity in the sensory analysis was possibly more due to the lactic acid added to the products to obtain color stability than the metabolism of starter and probiotic cultures. The color, together with texture, was pointed out as the characteristic most appreciated by the tasters. The dairy desserts maintained their stable color from the pigment (anthocyanins) of the jabuticaba peel due to the addition of lactic acid, as mentioned previously, as well as the use of the natural carmine dye that gave an attractive aspect for consumption. Anthocyanins exhibit low stability in fermented dairy products, sometimes influenced by storage temperature, pH and microbial cultures properties. Torskangerpoll and Andersen [51] have observed that pH is one of the main factors affecting the color of anthocyanin. In acid solutions, anthocyanin is red, but with increasing pH the color intensity decreases. This is the reason why lactic acid was used to obtain a more stable color in the present study. Compared with the results for probiotic dairy products from other studies, including commercial products, the scores obtained for the desserts of this study were considered to be satisfactory. 

## 6. Conclusions

The viability of the indigenous culture of *L. plantarum* CNPC003 remained above 7 log CFU per mL or g during the fermentation of the whey-enriched dairy bases and also throughout the storage of the fermented desserts with the jabuticaba peel ingredients. The probiotic candidate also did not change the mean composition, physical-chemical characteristics, total phenolic content, antioxidant capacity and instrumental texture dessert in comparison to the control and commercial probiotic trials. The desserts studied were characterized as low-fat products, presenting as acceptable for overall consumption, with an attractive color and appreciable texture, despite the acidic taste. The dessert with *L. plantarum,* in particular, could be considered a viable alternative for health promotion due to the concentration of lactobacilli comparable to that obtained for the commercial culture, besides the jabuticaba peel, which enriched the product with antioxidant phenolic compounds, and the use of whey, also contributing to a sustainable and healthy product.

## Figures and Tables

**Table 1:** Ingredients used to produce fermented dairy dessert of T1, T2 and T3 trials in this study.

			Trials	
Product Step	Ingredient (g)	T1	T2	T3
	Whey	840.0	840.0	840.0
	Sucrose	80.0	80.0	80.0
Dairy base	Milk powder	80.0	80.0	80.0
	*Streptococcus thermophilus* TA40	0.03	0.03	0.03
	*Lactobacillus rhamnosus* LR32	−	0.2	−
	*Lactobacillus plantarum* CNPC003	−	−	*
	Sum	1000.03	1000.23	1000.03 **
	Ingredient (g)			
	Fermented dairy base	900.0	900.0	900.0
	Jabuticaba peel syrup	57.655	57.655	57.655
Dessert mixture	Hydroethanolic extract of jabuticaba peel	20.0	20.0	20.0
	Pectin	17.5	17.5	17.5
	Lactic acid	4.8	4.8	4.8
	Cochineal carmine dye	0.450	0.450	0.450
	Sum	1000.405	1000.405	1000.405
	Ingredient (g)			
Final product	Dessert mixture	75.0	75.0	75.0
	Jabuticaba peel jam	12.5	12.5	12.5
	Sum	87.5	87.5	87.5

T1 = control, with the starter culture of *Streptococcus thermophilus* TA40; T2 = commercial probiotic, with *S. thermophilus* TA40 and the potentially probiotic commercial culture of *L. rhamnosus* LR32; T3 = experimental, produced with *S. thermophilus* TA40 and the indigenous *L. plantarum* CNPC003 culture; − = Ingredients not added. * = Added after harvesting 10 mL MRS growth culture by centrifugation, with discarding of the supernatant and washing culture three times with sterile saline solution. ** = Included the harvested *L. plantarum* CNPC003 culture.

**Table 2:** Mean composition of fermented dairy dessert trials containing jabuticaba peel ingredients on the first day of refrigerated storage at 4 ± 1 °C.

		Trials	
Parameter	T1	T2	T3
Total solids (g 100 g^−1^)	28.90 ± 2.74 ^A^	26.49 ± 4.98 ^A^	29.66 ± 8.92 ^A^
Ash—FW (g 100 g^−1^)	0.922 ± 0.225 ^A^	1.02 ± 0.72 ^A^	0.874 ± 0.132^A^
Ash—DM (g 100 g^−1^)	3.16 ± 0.56 ^A^	3.75 ± 2.49 ^A^	3.35 ± 1.69 ^A^
Fat—FW (g 100 g^−1^)	0.302 ± 0.145 ^A^	0.320 ± 0.145 ^A^	0.410 ± 0.169 ^A^
Fat—DM (g 100 g^−1^)	1.09 ± 0.62 ^A^	1.33 ± 0.82 ^A^	1.69 ± 1.30 ^A^
Protein—FW (g 100 g^−1^)	2.31 ± 0.33 ^A^	2.29 ± 0.36 ^A^	2.33 ± 0.42 ^A^
Protein—DM (g 100 g^−1^)	8.01 ± 1.09 ^A^	8.77 ± 1.33 ^A^	9.03 ± 5.17 ^A^
Total carbohydrate—FW (g 100 g^−1^)	25.37 ± 2.57 ^A^	22.86 ± 4.61 ^A^	26.04 ± 8.82 ^A^
Total carbohydrate—DM (g 100 g^−1^)	87.74 ± 1.19 ^A^	86.15 ± 3.16 ^A^	85.92 ± 7.99 ^A^

T1 = control, without lactobacilli adjunct; T2 = commercial probiotic, with *L. rhamnosus* LR32; T3 = experimental probiotic, with the indigenous culture of *L. plantarum* CNPC003. FW = fresh weight sample. DM = dry matter basis. ^A^ In a row, trials sharing the same superscript uppercase letter did not significantly differ for the same parameter (*p* > 0.05).

**Table 3:** Changes in mean values of pH, titratable acidity and in populations of *S. thermophilus* and *Lactobacillus* sp*.* of the fermented dairy dessert trials containing jabuticaba peel ingredients after 1, 7, 14 and 21 days of storage at 4 ± 1 °C.

	Time		Trial	
Parameter	(days)	T1	T2	T3
	1	3.88 ± 0.10 ^Aa^	3.87 ± 0.07 ^Aa^	3.97 ± 0.08 ^Aa^
pH	7	3.86 ± 0.18 ^Aa^	3.83 ± 0.10 ^Aa^	3.91 ± 0.12 ^Aa^
	14	3.87 ± 0.18 ^Aa^	3.90 ± 0.11 ^Aa^	3.97 ± 0.10 ^Aa^
	21	3.89 ± 0.11 ^Aa^	3.87 ± 0.06 ^Aa^	3.96 ± 0.10 ^Aa^
				
	1	1.11 ± 0.12 ^Aa^	0.99 ± 0.17 ^Aa^	1.06 ± 0.14 ^Aa^
Titratable acidity	7	1.13 ± 0.20 ^Aa^	1.12 ± 0.18 ^Aa^	1.11 ± 0.20 ^Aa^
(lactic acid 100 g^−1^)	14	1.06 ± 0.15 ^Aa^	1.03 ± 0.26 ^Aa^	1.02 ± 1.17 ^Aa^
	21	1.03 ± 0.30 ^Aa^	0.99 ± 0.23 ^Aa^	0.95 ± 0.26 ^Aa^
				
	1	9.26 ± 0.21 ^Ac^	9.07 ± 0.19 ^Ab^	9.39 ± 0.55 ^Ab^
*S. thermophilus*	7	8.97 ± 0.03 ^Ab^	8.92 ± 0.10 ^Aa^	8.99 ± 0.16 ^Aa^
(log CFU g^−1^)	14	8.94 ± 0.16 ^Aab^	8.92 ± 0.12 ^Aa^	8.93 ± 0.22 ^Aa^
	21	8.90 ± 0.06 ^Aa^	8.89 ± 0.02 ^Aa^	8.96 ± 0.20 ^Aa^
				
	1	n.a.	7.93 ± 0.10 ^Bc^	7.39 ± 0.59 ^Ac^
*Lactobacillus* sp.	7	n.a.	8.02 ± 0.31 ^Bc^	7.20 ± 0.52 ^Ab^
(log CFU g^−^^1^)	14	n.a.	7.70 ± 0.12 ^Bb^	7.01 ± 0.42 ^Aa^
	21	n.a.	7.43 ± 0.24 ^Aa^	7.12 ± 0.57 ^Aab^

T1 = control, without lactobacilli adjunct; T2 = commercial probiotic, with *L. rhamnosus* LR32; T3 = experimental probiotic, with the indigenous culture of *L. plantarum* CNPC003. n.a. = Not added. ^A, B^ = In a row, different superscript uppercase letters denote significant differences between trials for the same storage day (*p* < 0.05). ^a, b, c^ = In a column, different superscript lowercase letters denote significant differences between the storage days for the same trial (*p* < 0.05).

**Table 4:** Total phenolic content, percent inhibition of DPPH radicals, EC_50_ and total antioxidant capacity of fermented dairy dessert trials containing jabuticaba peel ingredients after 1, 7, 14 and 21 days of storage at 4 ± 1 °C.

Parameters	Trial	Time (days)			
		1	7	14	21
Total phenolics (mg GAE 100 g^−1^)	T1	54.10 ± 9.27 ^Aa^	64.52 ± 19.23 ^Aa^	59.09 ± 15.35 ^Aa^	62.77 ± 21.60 ^Aa^
	T2	45.08 ± 9.97 ^Aa^	58.36 ± 20.34 ^Aa^	56.09 ± 14.09 ^Aa^	61.60 ± 10.14 ^Aa^
	T3	50.03 ± 18.63 ^Aa^	56.71 ± 12.78 ^Aa^	68.93 ± 11.65 ^Aa^	56.56 ± 14.92 ^Aa^
Inhibition of radicals DPPH (%) *	T1	36.92 ± 5.74 ^Aa^	35.31 ± 10.80 ^Aa^	34.96 ± 12.03 ^Aa^	42.34 ± 5.18 ^Aa^
	T2	44.84 ± 2.35 ^Aa^	43.32 ± 1.40 ^Aa^	32.60 ± 0.78 ^Aa^	35.66 ± 9.06 ^Aa^
	T3	40.45 ± 2.30 ^Aa^	39.08 ± 0.99 ^Aa^	33.75 ± 2.08 ^Aa^	35.50 ± 1.46 ^Aa^
EC_50_ (g sample L^−1^ sol. DPPH 100 µM)	T1	5.30 ± 0.10 ^Aa^	6.32 ± 0.78 ^Aa^	5.76 ± 1.04 ^Aa^	5.61 ± 1.48 ^Aa^
	T2	4.94 ± 0.54 ^Aa^	4.54 ± 0.53 ^Aa^	4.22 ± 0.82 ^Aa^	6.15 ± 0.52 ^Aa^
	T3	4.60 ± 0.27 ^Aa^	4.49 ± 0.27 ^Aa^	5.27 ± 0.18 ^Aa^	5.46 ± 2.34 ^Aa^
Total antioxidant capacity (g sample g^−1^ DPPH)	T1	248.88 ± 7.39 ^Aa^	258.20 ± 5.47 ^Aa^	229.11 ± 21.97 ^Aa^	239.76 ± 84.74 ^Aa^
	T2	205.56 ± 8.03 ^Aa^	207.10 ± 10.23 ^Aa^	165.77 ± 48.07 ^Aa^	237.36 ± 13.39 ^Aa^
	T3	234.87 ± 29.23 ^Aa^	210.27 ± 21.51 ^Aa^	253.78 ± 8.52 ^Aa^	248.20 ± 108.85 ^Aa^

DPPH = 1,1-diphenyl-2-picrylhydrazyl; EC_50_ = amount of sample required to reduce the initial concentration of DPPH by 50%; T1 = control, without lactobacilli adjunct; T2 = commercial probiotic, with *L. rhamnosus* LR32; T3 = experimental probiotic, with the indigenous culture of *L. plantarum* CNPC003. * Values for the maximum sample concentration used in the assay: 200 μL of sample extract added to 2,800 μL of 100 μM DPPH solution (3 mL total volume). ^A^ In a column, trials sharing the same superscript uppercase letter did not differ significantly in the same storage period for the same parameter (*p* > 0.05). ^a^ In a row, storage days sharing the same superscript lowercase letter did not differ significantly considering the same formulation (*p* > 0.05).

**Table 5:** Instrumental firmness, consistency, cohesiveness* and viscosity index* of fermented dairy dessert trials containing jabuticaba peel ingredients after 1, 7, 14 and 21 days of storage at 4 ± 1 °C.

Parameters	Treatments	Time (days)			
		1	7	14	21
Firmness (N)	T1	0.596 ± 0.24 ^Aa^	1.26 ± 0.37 ^Ab^	0.662 ± 0.23 ^Aa^	0.811 ± 0.27 ^Aa^
	T2	0.655 ± 0.20 ^Aa^	0.813 ± 0.21 ^Aa^	0.836 ± 0.28 ^Aa^	0.958 ± 0.38 ^Aa^
	T3	0.885 ± 0.38 ^Aa^	1.01 ± 0.22 ^Aa^	0.770 ± 0.14 ^Aa^	0.90 ± 0.26 ^Aa^
Consistency (N × s)	T1	8.35 ±3.97 ^Aa^	12.55 ± 5.89 ^Ab^	9.70 ± 4.68 ^Aa^	11.35 ± 4.05 ^Ab^
	T2	9.41 ± 3.13 ^Aab^	12.74 ± 4.96 ^Ab^	10.87± 4.03 ^Ab^	8.79 ± 5.79 ^Aa^
	T3	9.41 ± 4.01 ^Aa^	13.46 ± 2.44 ^Ab^	12.82 ±2.82 ^Aab^	11.17 ± 2.67 ^Aa^
Cohesiveness (N)	T1	0.515 ± 0.24 ^Ba^	0.838 ± 0.11 ^Cb^	0.435 ± 0.47 ^Aa^	0.555 ± 0.29 ^Aa^
	T2	0.338 ± 0.09 ^Aa^	0.516± 0.19 ^Ab^	0.499 ± 0.19 ^Ab^	0.574 ± 0.34 ^Ab^
	T3	0.505 ± 0.20 ^Ba^	0.689 ± 0.09 ^Bb^	0.560 ± 0.17 ^Aab^	0.604± 0.09 ^Aab^
Viscosity index (N × s)	T1	0.499 ± 0.33 ^Aa^	0.884 ± 0.57 ^Abc^	0.769 ± 0.27 ^Ab^	1.015 ± 0.39 ^Ac^
	T2	0.624 ± 0.26 ^Aa^	0.869 ± 0.38 ^Aa^	0.801 ± 0.34 ^Aa^	0.811 ± 0.41 ^Aa^
	T3	1.186 ± 0.32 ^Bb^	1.290 ± 0.12 ^Ab^	0.978 ± 0.23 ^Aa^	0.953 ± 0.11 ^Aa^

T1 = control, without lactobacilli adjunct; T2 = commercial probiotic, with *L. rhamnosus* LR32; T3 = experimental probiotic, with the indigenous culture of *L. plantarum* CNPC003. ^A,B,C^ = In a column, different superscript uppercase letters denote significant differences between trials for the same storage day (*p* < 0.05). ^a,b,c^ = In a row, different superscript lowercase letters denote significant differences between the storage days for the same trial (*p* < 0.05).

**Table 6:** Sensory attributes cited as “most appreciated” and “less appreciated” by the judges (*n* = 35) for of fermented dairy dessert trials containing jabuticaba peel ingredients on Days 7 and 21 of storage.

Trial	Ranking	Time (days)	Attributes Cited				Total Citations *n* (%)
			Flavor *n* (%)	Texture *n* (%)	Appearance *n* (%)	Color *n* (%)	
T1	Most	7	9 (26)	13 (37)	2 (6)	11 (31)	35 (100)
	appreciated	21	11 (31)	7 (20)	5 (14)	12 (34)	35 (100)
	Less	7	20 (57)	3 (9)	4 (11)	8 (23)	35 (100)
	appreciated	21	19 (56)	3 (9)	5 (15)	7 (21)	34 (100)
T2	Most	7	10 (29)	10 (29)	6 (17)	9 (26)	35 (100)
	appreciated	21	8 (22)	10 (28)	6 (17)	12 (33)	36 (100)
	Less	7	16 (46)	7 (20)	2 (6)	10 (29)	35 (100)
	appreciated	21	21 (60)	5 (14)	3 (9)	6 (17)	35 (100)
T3	Most	7	9 (26)	10 (29)	3 (9)	13 (37)	35 (100)
	appreciated	21	8 (23)	7 (20)	6 (17)	14 (40)	35 (100)
	Less	7	22 (61)	4 (11)	7 (19)	3 (8)	36 (100)
	appreciated	21	16 (46)	11 (31)	2 (6)	6 (17)	35 (100)

T1 = control, without lactobacilli adjunct; T2 = commercial probiotic, with *L. rhamnosus* LR32; T3 = experimental probiotic, with the indigenous culture of *L. plantarum* CNPC003.
